# Network mechanisms of intentional learning

**DOI:** 10.1016/j.neuroimage.2015.11.060

**Published:** 2016-02-15

**Authors:** Adam Hampshire, Peter J. Hellyer, Beth Parkin, Nole Hiebert, Penny MacDonald, Adrian M. Owen, Robert Leech, James Rowe

**Affiliations:** a The Computational, Cognitive and Clinical Neuroimaging Laboratory, Division of Brain Sciences, Imperial College London, London, UK; b Centre for Neuroimaging Sciences, Institute of Psychiatry, Psychology and Neuroscience, King's College London, UK; c Institute of Cognitive Neuroscience, University College London, London, UK; d The Brain and Mind Institute, University of Western Ontario, London, ON, Canada; e Department of Clinical Neurosciences, University of Cambridge, Cambridge, UK; f Medical Research Council Cognition and Brain Sciences Unit, Cambridge, UK

**Keywords:** Learning, Frontal cortex, Caudate, Functional connectivity, Dynamic causal modelling

## Abstract

The ability to learn new tasks rapidly is a prominent characteristic of human behaviour. This ability relies on flexible cognitive systems that adapt in order to encode temporary programs for processing non-automated tasks. Previous functional imaging studies have revealed distinct roles for the lateral frontal cortices (LFCs) and the ventral striatum in intentional learning processes. However, the human LFCs are complex; they house multiple distinct sub-regions, each of which co-activates with a different functional network. It remains unclear how these LFC networks differ in their functions and how they coordinate with each other, and the ventral striatum, to support intentional learning. Here, we apply a suite of fMRI connectivity methods to determine how LFC networks activate and interact at different stages of two novel tasks, in which arbitrary stimulus-response rules are learnt either from explicit instruction or by trial-and-error. We report that the networks activate *en masse* and in synchrony when novel rules are being learnt from instruction. However, these networks are not homogeneous in their functions; instead, the directed connectivities between them vary asymmetrically across the learning timecourse and they disengage from the task sequentially along a rostro-caudal axis. Furthermore, when negative feedback indicates the need to switch to alternative stimulus–response rules, there is additional input to the LFC networks from the ventral striatum. These results support the hypotheses that LFC networks interact as a hierarchical system during intentional learning and that signals from the ventral striatum have a driving influence on this system when the internal program for processing the task is updated.

## Highlights

• We examine lateral frontal cortex (LFC) network dynamics across the learning process.
• LFC networks activate *en masse* and in synchrony when learning from instruction.
• Directed connectivities between LFC regions vary asymmetrically and hierarchically.
• LFC networks sequentially disengage from the task along an anterior–posterior axis.
• Corticostriatal circuits are selectively engaged during trial-and-error learning.

## Introduction

Humans have a remarkable ability to learn new tasks rapidly. We often perform them near flawlessly based on instruction, observation, mental simulation, or the outcomes of individual attempts. These intentional forms of learning involve flexible cognitive systems, which rapidly adapt to encode temporary programs for processing non-automated tasks in a controlled manner.

**Fig. 1:**
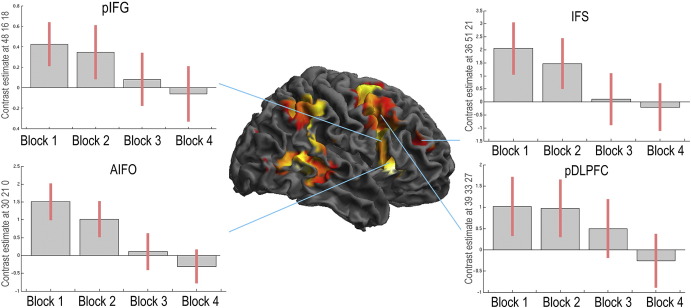
Paradigms that are used to probe LFC function often treat learning effects as nuisance variables. This can lead to overly static interpretations of LFC function. For example, one prominent hypothesis states that a sub-region of the right inferior frontal gyrus (pIFG) is involved in the effortful cancellation of dominant motor responses. The Stop Signal Task is designed to probe motor inhibition processes and shows significant activation within this region. However, the pIFG is most active when the task is initially being learnt. Other LFC sub-regions, including the anterior insula inferior frontal operculum (AIFO), inferior frontal sulcus (IFS) and posterior dorsolateral prefrontal cortex (pDLPFC), show similar learning effects. Behavioural performance measures correlate with changes in functional connectivity between these LFC sub-regions. These results (Erika-Florence et al., 2014) indicate that distributed LFC networks work in a coordinated manner to support novel tasks. As a task becomes automated, the involvement of these networks diminishes.

There is a wealth of evidence for the role of the lateral frontal cortex (LFC) in coding for these temporary programs (Duncan, 2001). For example, at the resolution of multi-unit electrophysiology, populations of neurons within the primate LFCs represent task-relevant information, including the stimuli, responses, and rules that constitute the task (Freedman et al., 2001, Miller and Cohen, 2001). They can adapt rapidly, switching from representing one aspect of a task to another in a fraction of a second (Stokes et al., 2013). At the regional-anatomical scale, neuropsychological research has shown that frontal lobe damage leads to cognitive inflexibility; that is, the inability to learn new behaviours or to override those that are habitual (Gaffan and Harrison, 1988, Halsband and Freund, 1990, Halsband and Passingham, 1982, Petrides, 1985, Petrides, 1990, Petrides, 1997). Furthermore, functional magnetic resonance imaging (fMRI) has demonstrated that the human LFCs are strongly activated during a variety of tasks that require the intentional control of thoughts and actions (Duncan and Owen, 2000, Fedorenko et al., 2013) including when tasks are being performed based on instructed rules (Rowe et al., 2007, Zhang et al., 2013). Most relevantly, when simple cognitive tasks are being performed in the scanner, the LFCs respond more at the beginning of the experiment, when stimulus–response rules are novel (Fig. 1), with little or no response towards the end, when they are routine (Boettiger and D'Esposito, 2005, Erika-Florence et al., 2014, Toni and Passingham, 1999, Toni et al., 2001).

**Fig. 2:**
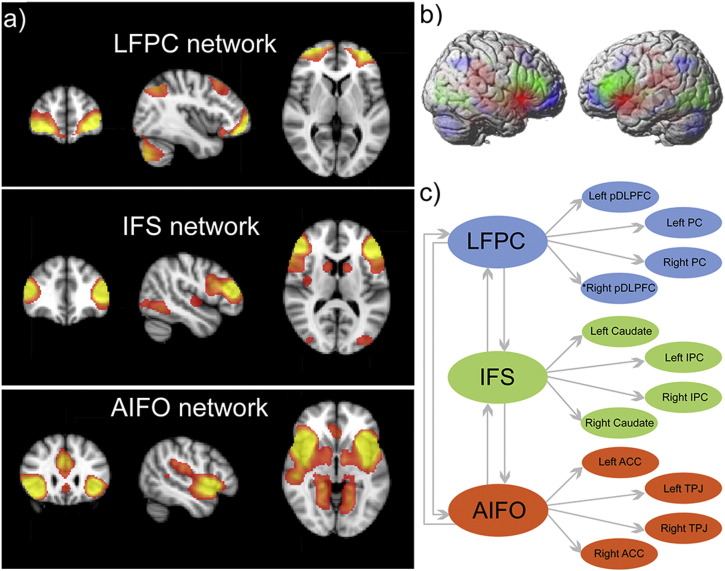
a) & b) In a previous study (Parkin et al., 2015) we applied spatial ICA to decompose the LFCs into functionally distinct sub-regions in a data-driven manner. The timecourses of these sub-regions were used in seed analyses to characterise their cortically and sub-cortically distributed functional networks. One network (red) included the AIFO. Seed analyses identified the anterior cingulate cortex/pre-supplementary motor area (ACC) and temporal parietal junction (TPJ) bilaterally. A second network (green) included the IFS. Seed analysis identified the caudate nucleus bilaterally and a region extending from the superior occipital lobe into the inferior parietal cortex (IPC). A third network (blue) included the lateral frontopolar cortices (LFPC). Seed analyses identified the pDLPFC and superior parietal cortices bilaterally (PC). c) 10 mm radius spherical regions of interest were defined based on peak coordinates from the ICA and seed analyses. These ROIs were formed into a two-tier network model, with the lower tier consisting of intra-network connections and the upper tier consisting of connections between different LFC sub-regions.

Although it is well established that the LFCs are involved in intentional learning, the mechanisms by which they interact and adapt are not yet fully understood. This is in part because the functional organisation of the human LFCs is often conceptually simplified to enable experimental tractability (Passingham and Wise, 2012). For example, classic studies focused on mapping functional dissociations across large-scale dorsal-ventral and anterior–posterior axes within the LFCs. However, data-driven analyses have shown that the LFCs are more complex than this (Hampshire and Sharp, 2015); they contain multiple, functionally distinct sub-regions, which each co-activate with a different large-scale connectivity network (Beckmann and Smith, 2004, Dosenbach et al., 2006, Dosenbach et al., 2008, Erika-Florence et al., 2014, Hampshire et al., 2012b, Laird et al., 2011, Smith et al., 2009). Three of these LFC networks (Fig. 2) include brain regions that are known to play particularly flexible roles in cognition (Duncan, 2001, Duncan and Owen, 2000, Fedorenko et al., 2013) and that are implicated in learning (Toni and Passingham, 1999, Toni et al., 2001). One network includes the anterior insular/inferior frontal operculum, the anterior cingulate cortex and the temporal-parietal junction bilaterally (AIFO network). Another includes the inferior frontal sulcus, the inferior parietal cortex and the ventral caudate bilaterally (IFS network). The third includes the lateral frontopolar cortex, the posterior dorsolateral prefrontal cortex and the superior parietal cortex bilaterally (LFPC network). It remains unclear how these networks differ in their functions and how they coordinate with each other to support controlled modes of behaviour such as intentional learning.

The ventral striatum has also been implicated in the learning of novel tasks and is richly connected to several LFC regions. However, it also reliably dissociates from the LFCs under some cognitive conditions (Hampshire et al., 2012a). For example, it has been reported that parameters from computational simulations of model-based and model-free reinforcement learning predict regional brain activations within the LFCs and the ventral striatum respectively (Glascher et al., 2010). More broadly, the ventral striatum has been implicated in the processing of task feedback, particularly reward prediction errors (O'Doherty et al., 2003, Schonberg et al., 2007, Seymour et al., 2004). Based on this, it has been proposed that the LFCs and the ventral striatum carry distinct learning signals (Glascher et al., 2010). However, less is known about how the LFCs and ventral striatum interact when these learning signals must be integrated: for example, when feedback signals the requirement to modify the temporary internal program for performing the task.

Here, we address these questions by applying a combination of fMRI analysis methods to examine LFC network activity and connectivity across consecutive stages of two stimulus–response learning tasks. First, we use a combination of precisely controlled contrasts and analyses of global network synchrony to test the hypothesis that LFC networks are more active and functionally interconnected during the simplest form of intentional learning, in which stimulus–response rules are applied based on explicit instruction at the start of each learning block with no reinforcement from feedback. Then, we use focused regions of interest (ROI) and psychophysiological interaction (PPI) analyses to test the hypothesis that striatocortical connections are engaged when the stimulus–response rules are being established based on feedback (O'Doherty et al., 2003, Schonberg et al., 2007, Seymour et al., 2004). Finally, we apply dynamic causal modelling (DCM) with Bayesian model selection to test whether LFC sub-regions interact in a hierarchical manner during learning from instruction and to examine how negative feedback impacts on striatocortical interactions during learning by trial and error.

## Materials and methods

### Participants

17 healthy participants (7 female and 10 male) aged 19–27 years completed Study 1 and 14 participants (5 female and 9 male) aged 20–35 years completed Study 2. All participants were right handed English speakers with normal or corrected to normal eyesight. Volunteers were excluded if they had a history of neurological or psychiatric illness, were taking psychoactive medications or did not meet MRI safety criteria. The local research ethics board approved this study. Participants gave informed consent prior to entering the fMRI scanner.

### Task designs

**Fig. 3:**
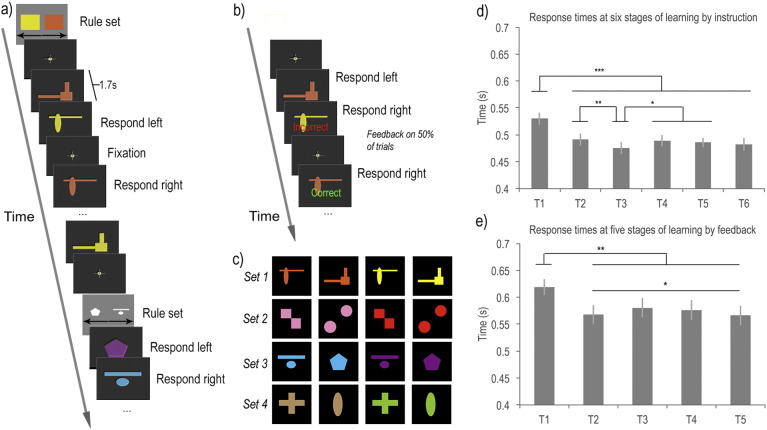
a) In Study 1 participants learnt novel discrimination rules from explicit instruction. Initially, a slide was presented with a discrimination rule. Subsequently, a sequence of coloured shapes was presented for 3 min and the participant was required to respond with the relevant button press as quickly and accurately as possible. After 3 min a new rule slide was displayed followed by another sequence of coloured shapes. b) In Study 2, there were no rule slides. Instead, feedback indicating whether the previous response was correct or incorrect was presented randomly after 50% of trials. Therefore, participants were required to derive the discrimination rules by a process of trial-and-error. c) The two studies used the same stimulus sets. d) In Study 1, response times followed a non-monotonic decrease when stimulus–response rules were being learnt from instruction. Specifically, there was a rapid decrease in RT from stages one to three, followed by a small increase in RT then a more gradual decrease. e) In Study 2, a similar non-monotonic decrease was also evident in RTs when stimulus-response rules were being learnt by exploration with feedback. (***p < 0.001, **p < 0.01, *p < 0.05 two tailed significance).

In Study 1 (Fig. 3a), participants were presented with a simple discrimination rule for 4 s (e.g. yellow shapes = left button response and orange shapes = right button response) followed by a sequence of coloured shapes. There were 4 compound stimuli per rule, constructed from 2 exemplars per dimension. There was no feedback post response. Stimuli were presented in randomised order at a rate of 1 per 1.7 s with 1/3 of trials showing fixation as opposed to a stimulus, which allowed activation during discriminations to be estimated relative to fixation. Presentation continued for 3 min, subsequent to which a new rule was presented; therefore, activations related to rule learning were not confounded by the total time spent in scanner or on task. Rules always changed across dimensions; i.e. if one rule related to shape then the next related to colour and all exemplars were replaced when the rules changed; this design ensured that the previously learned stimulus–response mappings did not have to be overridden. There were a total of four rule slides, each followed by a 3 min sequence of discriminations. Study 2 (Fig. 3b) used a variant on the design of Study 1 with the same rules and stimuli (Fig. 3c). However, the participants had to derive the rule based on feedback as opposed to explicit instruction with a rule slide. Feedback was presented centrally on the screen as either the word ‘Correct’ in green or ‘Incorrect’ in red after a random 50% of trials, which allowed activations related to rule novelty and feedback to be estimated separately. The duration of each block was reduced to 2.5 min based on the rapid learning effects observed in Study 1. Behavioural outlier values (defined as > 2.5 SDs from the mean) were winsorised within condition for both studies to ensure they did not distort the results.

### Data acquisition

Responses were made on an MRI compatible button box using the index and middle fingers of the right hand. Tasks were programmed in Visual Basic and stimuli were projected on a screen, visible via a mirror, at the end of the scanner bore. Brain images were collected using a 3 Tesla Siemens Scanner. A T2 weighted echo planar image depicting blood oxygenation level dependent (BOLD) contrast was acquired every 2 s. The first 10 images were discarded to account for equilibrium effects. Images consisted of 32 ∗ 3 mm slices, with a 64 × 64 matrix, 192 × 192 mm field of view, 30 ms TE, 2 s TR, 78° flip angle, 0.51 ms echo spacing, and 2232 Hz/Px bandwidth. A 1 mm resolution MPRAGE structural scan was also collected for each individual with a 256 × 240 × 192 matrix, 900 ms TI, 2.99 ms TE and 9° flip angle. Data were pre-processed using a standard pipeline in SPM8 (Statistical Parametric Mapping, Welcome Department of Imaging Neuroscience). Specifically, they were slice-timing and motion corrected, spatially warped onto the standard Montreal Neurological Institute template using the structural scan, and spatially smoothed with an 8 mm full width at half maximum Gaussian kernel.

### Univariate analysis

FMRI data were analysed at the individual participant level in SPM8 using general linear models (GLMs). In Study 1, discrimination trials were modelled using six predictor functions, each consisting of event timings convolved with the canonical haemodynamic response function. These included the onsets and durations of all discrimination trials broken down into 6 × 30 second ‘learning stages’ arranged contiguously to estimate neural activation at a coarse grain as rules transitioned from novel to familiar. For example, the first predictor included all discriminations from the first 30 s after definition of each of the four rules. The second predictor captured trials within the next 30 s, etc. A seventh predictor captured the onset and duration of the four rule definition events. Six additional predictors were included to capture noise due to head movements. These were the translations and rotations in the x, y and z planes.

Event-related fMRI data for Study 2 were modelled in the same manner as Study 1 with the following exceptions. Onsets and durations for stimuli with responses were again broken down into 30-second learning stages; however, they formed 5 predictor functions as each learning block (i.e. period of time when the rule was applied) was 2.5 as opposed to 3 min long. Also, in Study 2 learning was driven by feedback as opposed to explicit instruction; therefore, the positive and negative feedback events were included in the model as two additional predictor functions and there was no rule definition predictor.

Whole brain maps depicting parameter estimates for the experimental predictor functions were exported for group level random effects analyses. Analyses of nodes within the LFC networks were conducted using focused regions of interest (ROIs) with the MarsBaR toolbox (Brett et al., 2002), which calculates the average value from all voxels within the ROI. ROIs (Fig. 2) were predefined in a recently reported study (Parkin et al., 2015) in the following manner. First spatial independent component analysis was conducted on a volume restricted to the lateral frontal cortices. This generated a detailed functional decomposition of the LFC volume. Then ROIs were defined at the peak bilateral coordinates for each component and their activation timecourses were extracted. The timecourses were regressed together onto each voxel in the brain. This seed analysis identified a set of brain regions that were representative of the networks that each LFC ROI co-activated with. Supplementary voxel-wise group level analyses were carried out in SPM8 and, unless reported otherwise, used cluster correction with initial voxelwise thresholding at p < 0.01 uncorrected followed by family wise error FWE cluster correction for the whole brain mass at p < 0.05.

### Functional connectivity analyses (undirected graphs)

Task-related changes in network connectivity were examined using two types of analyses, each of which provides a different insight into network interactions. First, phase synchrony analyses were applied to timecourse data extracted from each ROI using MarsBaR. Notably, unlike more established fMRI functional connectivity methods (e.g. psychophysiological interactions), phase synchrony analysis scales efficiently with the number of reciprocal connections, thereby allowing a global connectivity timecourse to be estimated from an entire set of network nodes. The phase synchrony timecourse may then be examined in relation to psychological conditions. This approach has proven sensitive to connectivity changes related to cognitive conditions in a previous study examining the same LFC networks (Parkin et al., 2015).

The timecourse data were high pass filtered at 60 s and an instantaneous measure of phase estimated by applying the Hilbert transform (Laird et al., 2002). Phase synchrony across time was then estimated using the Kuramoto Order parameter (Hellyer et al., 2014). Essentially, a timecourse representing the phase synchrony was calculated by taking the exponent of the phase multiplied by the square root of − 1 for each data point, providing a complex representation with magnitude of one and argument dependent on phase angle. The absolute of the mean of this representation across the timecourses at each time point, provides a convenient measure of phase co-ordination:

$$R\left(t\right)=\frac{1}{N}\sum_{n=1}^{N}e^{i\Theta_{n}\left(t\right)}$$

where R is a vector representing the level of phase synchrony between *N* timecourses (ROIs or voxels) at each time point (t) and *Θ* represents the *N* ∗ *t* matrix of instantaneous phases. 1 = fully synchronous and 0 = fully asynchronous timecourses. The model from the participant's SPM GLM, including all psychological events, movement parameters and the constant term, was regressed onto the synchrony timecourse R. Parameter estimates from the regression model were collated for group level analysis to determine whether there were consistent task-related changes in connectivity across the networks. This analysis was repeated with timecourses extracted from all voxels within the brain in order to determine whether the learning manipulations evoked a global change in low frequency synchrony.

Next, psychophysiological interaction (PPI) analyses were carried out (Friston et al., 1997) between individual pairs of regions in order to determine whether task-related changes in functional connectivity differed for specific network connections. PPIs were conducted using SPM8 in the following standard manner. BOLD activation timecourses were extracted from bilateral masks composed of 10 mm radius spheres within the seed region using the Volume of Interest (VOI) function, which extracts the first eigenvector across all voxels within the ROI. The neural signal underlying the BOLD response was estimated using the SPM deconvolution function prior to being interacted with psychological timecourses to produce the PPIs. The physiological, psychological and psychophysiological timecourses were re-convolved with the canonical hemodynamic response function to produce a set of three predictors. These, together with movement parameters, were fitted with a GLM onto each target ROI. Mean parameter estimates for the PPI predictors were extracted for each seed-target PPI model using the MarsBaR ROI toolbox. These data were exported for group-level analyses in SPSS.

### Effective connectivity analysis (directed graphs)

Dynamic causal modelling was conducted in SPM12 using bilinear deterministic models (Friston et al., 2003). These generative models for fMRI use Bayesian model inversion to optimise both neuronal interactions and the neurovascular forward model (hrf) at each region to maximise the log-model evidence (accuracy, corrected for complexity). In Study 1, each model was fitted to time-course data from three ROIs. Timecourses were extracted using the VOI function from masks composed of bilateral 10 mm radius spheres based at the peak coordinates from the ICA for the three LFC ROIs (Fig. 2). The ROIs were reciprocally connected and self connected (A matrix). The driving input for all models (C matrix) was a Task contrast that included the durations for all events at all learning stages (stimulus–response events during Stages 1–6 all weighted as 1). Models varied with respect to the target of the modulatory input (B matrix), which was a Novelty contrast (entire duration of Stages 1–6 weighted as 3 2 1 − 1 − 2 − 3). The most optimal model was selected using Bayesian model selection with fixed effects analysis. The fixed effects approach is most appropriate because one can assume that healthy controls have consistent network architecture (Stephan et al., 2010). However, it should be noted that Bayesian model selection with random effects analysis also favoured the same models. In Study 2, all models built on the optimal model from Study 1 analysis with the modulatory input adapted to account for the reduced number of learning stages (duration of Stages 1–5 = 2 1 0 − 1 − 2). An additional ventral striatum region was included, consisting of the caudate ROIs bilaterally and a timecourse of negative feedback events formed an additional input to the models. The models varied in terms of whether feedback was a driving or modulatory input and the nodes/connections that this input targeted.

## Results

### Behavioural analyses

In Study 1 (learning from instruction), performance measures were averaged across sequences and within each of six consecutive thirty-second stages. (Averaging in this manner was valid as there was no significant difference between the response time measures of the four sequences). Participants performed the task with > 97% accuracy at all stages. Repeated measures analysis of variance (ANOVA) with Stages 1–6 as the factor showed a significant learning effect with RTs decreasing as the discriminations became routine (F_(5,75)_ = 15.13 p < 0.001). Mean RTs did not reduce monotonically with learning; instead, Stage 3 showed a small early minimum. Post-hoc analysis of just the latter five stages confirmed this effect with a significant cubic within-subject contrast (F_(1,15)_ = 4.54 p = 0.005). Pairwise t-tests confirmed that Stage 3 RTs were significantly faster than those of Stages 1, 2, 4 or 5 whilst Stage 1 was slower than all five others (Fig. 3d).

**Fig. S1:**
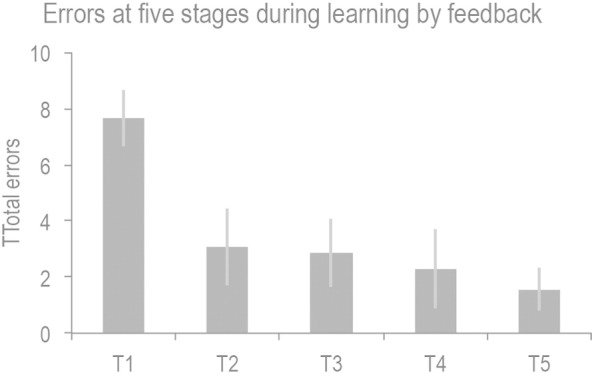
Mean number of incorrect responses made at each of the five stages of learning by trial and error. Error bars report the standard error of the mean.

In Study 2, (learning from feedback) performance measures were sorted into five consecutive 30-second stages (Stages 1–5). Participants received an average of 18.3 negative feedback events across the session (Inline Supplementary Fig. S1). A repeated measures ANOVA with Stage (1–5) as the factor showed a significant main effect of stage on errors (F_(4,52)_ = 20.254 p < 0.001). The response time data were examined with a further repeated measures ANOVA with Stages 1–5 as the factor. There was a significant reduction in RTs with learning (F_(4.52)_ = 9.01 p < 0.001) and a similar non-monotonic decrease in RTs to Study 1; however, the minimum occurred earlier, in Stage 2. Post-hoc analysis of the latter four stages indicated a significant quadratic within-subject contrast (F_(1,13)_ = 5.703 p = 0.033). Pairwise comparisons showed significant differences between the RTs in Stage 1 and all subsequent stages (Fig. 3e). However, the non-monotonic effect was subtler than for Study 1, because Stage 2 did not differ significantly from Stages 3–5 in the pairwise comparisons.

Inline Supplementary Figure S1

### Analysis of activation magnitudes: between-network comparison of the effects of learning

**Fig. S2:**
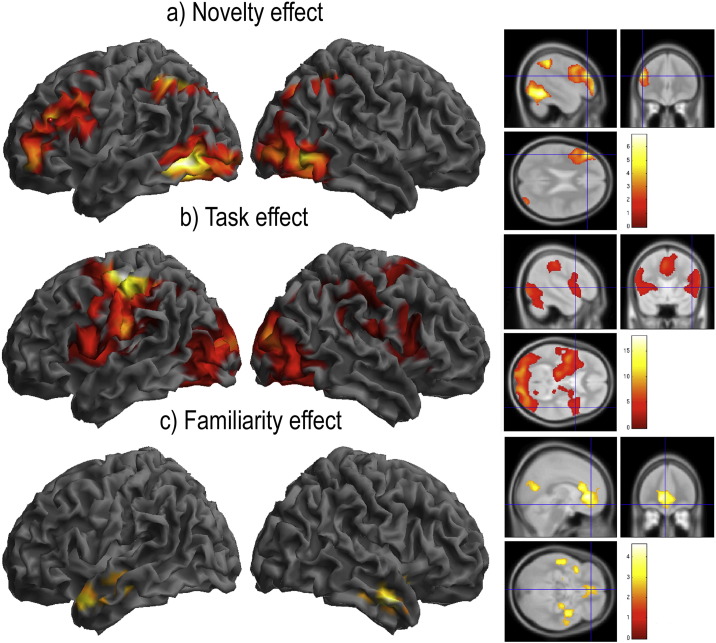
Supplemental whole-brain analyses of data from study 1 (all rendered at p < 0.05 FWE cluster corrected for the whole brain volume). Voxelwise analysis using a novelty contrast during learning by instruction (Stages 1–6 weighted as 3 2 1 − 1 − 2 − 3) rendered significant effects across a set of brain regions including the left IFS and the IPC bilaterally (S2a). A Task contrast (Stages 1–6 all weighted as 1) rendered activation across a set of brain regions including the AIFO and ACC (S2b). The negative effect of Novelty rendered significant voxels within brain regions that approximately conform to the default mode network (Buckner et al., 2008) (S2c). There were no significant voxels within the striatum for any of the above contrasts even at the uncorrected threshold of p < 0.05 one-tailed.

**Fig. S3:**
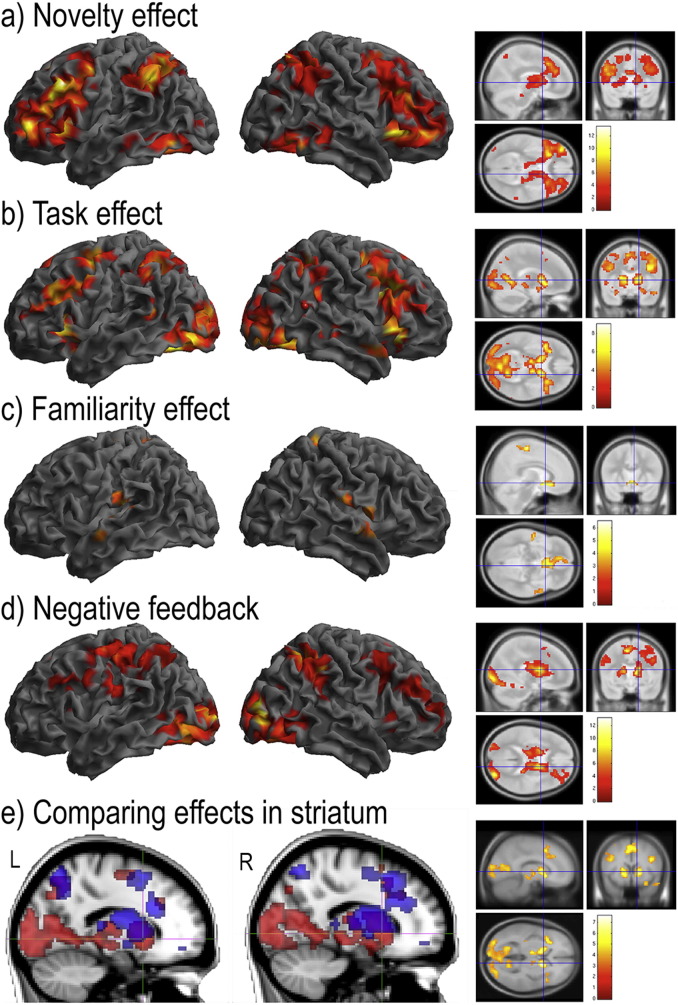
Supplemental whole-brain analyses of data from Study 2 (all rendered at p < 0.05 FWE cluster corrected for the whole brain volume). Voxelwise analysis using a Novelty contrast during learning from feedback (Stages 1–5 weighted as 2 1 0 − 1 − 2) rendered significant activation across the frontoparietal cortices and within the striatum (S3a). A similar pattern of activation was evident for the Task contrast (Stages 1–5 all weighted as 1) (S3b). A set of regions within the medial orbitofrontal cortices and temporal lobes were more active when rules were familiar (S3c). Negative feedback generated strong activation within the frontoparietal cortices and within the striatum (S3d). Notably, peak coordinates within the caudate varied between the contrasts (S3e), with mid/ventral regions including the nucleus accumbens, putamen and ventral caudate activated by negative feedback (rendered in red on the sagittal images) but not Novelty (rendered in blue on the sagittal images). Comparing directly between these contrasts (inset on the right) showed that the feedback-familiarity difference was significant at the whole brain corrected threshold, whereas the reverse contrast was not significant even at the uncorrected threshold of p < 0.05. The peak striatum coordinates for this contrast of contrasts (left x = − 12 y = 5 z = 4; right x = 15 y = 8 z = 4) were within 5 mm of the centres of the caudate ROIs (left x = − 12 y = 10 z = 4; right x = 12 y = 12 z = 4).

**Fig. 4:**
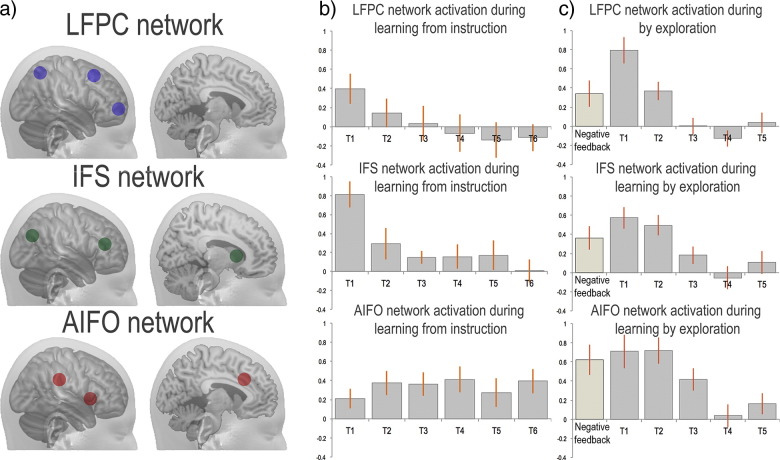
a) Placement of the LFC network regions of interest. b) The IFS and LFPC networks showed smooth downwards curves in task-related activation as the stimulus–response rules transitioned from novel to familiar. By contrast, the AIFO network still showed significant task-related activation at the sixth and final stage of the learning process. c) All three networks showed decreases in task-related activation as the rules transitioned from novel to familiar during learning by exploration with feedback. The LFPC showed the sharpest decline, with a large early peak, whereas the AIFO showed the slowest decline, with significant activation through the third stage. The networks also showed significant activation during negative feedback (left bar).

Analyses of regional activation magnitudes focused on the two-tier network model (Fig. 4a). For completeness, voxelwise analyses are reported in the inline supplemental materials (Inline Supplementary Figure S2, Inline Supplementary Figure S3). Activities of the three LFC networks were examined across the six stages of Study 1 to determine whether they differed in their sensitivities to learning from instruction. Parameter estimates were averaged across all ROIs for each network at each learning stage and the resultant data were examined using repeated measures ANOVA with the conditions Stage 6 and Network (3). There was a significant positive effect of condition (T contrast averaged across conditions) indicating that the LFC networks were generally more active during task than at rest (t = 3.709 p < 0.001 one tailed). There were significant main effects of Stage (F_(5,80)_ = 2.4 p = 0.044) and Network (F_(2,32)_ = 4.632 p = 0.017), and a significant Stage ∗ Network interaction (F_(10,160)_ = 4.787 p < 0.001), indicating that the networks had different sensitivities to learning from instruction. When the mean network activation levels were plotted for each stage to determine the basis of the interaction (Fig. 4b), the IFS and LFPC networks followed a smooth decline in activation with learning whereas the AIFO network showed consistent activation across the learning stages.

Inline Supplementary Figure S2

Inline Supplementary Figure S3

Network activities were then examined across the five stages of learning by trial and error. Parameter estimates were averaged across all ROIs for each network and examined using repeated-measures ANOVA with the conditions Stage 5 and Network (3). There was a significant positive effect of condition (t = 4.828 p < 0.001), and a significant main effect of Stage (F_(4,52)_ = 13.795 p < 0.001) but not of Network (F_(2,26)_ = 2.062 p = 0.147). There was a significant Stage ∗ Network interaction (F_(8,104)_ = 4.647 p < 0.001), indicating that the networks had different sensitivities to learning from feedback. When the parameter estimates were plotted separately at each stage of the task (Fig. 4c) it was evident that the activation response to discriminations declined most rapidly for the LPFC network, which showed a strong peak in the first stage and least rapidly for the AIFO network, which was active in the third learning stage.

### Within-network comparison of the effects of learning

**Fig. 5:**
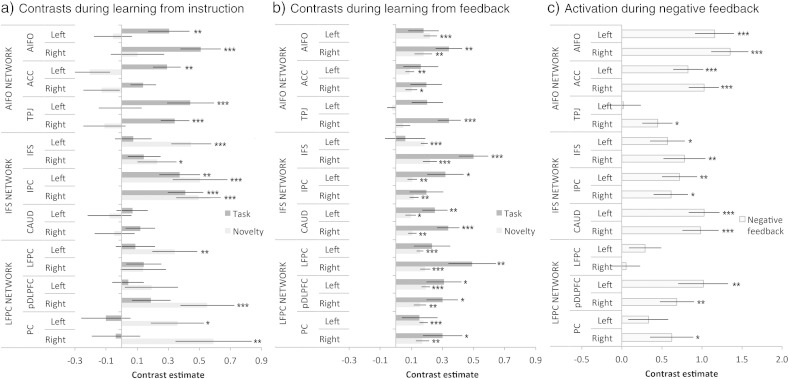
a) Task and Novelty effects for each region of interest during learning from instruction. Notably, the caudate ROIs showed no significant effects of task or novelty whereas the rest of the IFS network was significantly activated by either one or both contrasts. b) During learning from feedback, there were significant effects of task relative to fixation and of rule novelty throughout all three networks. In contrast to learning by instruction, these effects were evident within the caudate (highlighted) and the AIFO network ROIs. c) Negative feedback generated heightened activation within all three networks including the caudate ROIs. (***p < 0.001, **p < 0.01, *p < 0.05 2-tailed significance. Error bars report the standard error of the mean).

Further analyses were conducted to determine whether there were differences in the sensitivities of the ROIs within each network to learning by instruction. A linear rule-novelty contrast was estimated across stages for each ROI (Stages 1–6 weighted as 3 2 1 − 1 − 2 − 3). Contrast estimates were examined in three separate one-way repeated measures ANOVAs in which the condition was ROI (6). There was a significant effect of ROI in the IFS network only (IFS network F_(5,80)_ = 4.84 p < 0.001; AIFO network F_(5,80)_ = 1.18 p > 0.3; LFPC network F_(5,80)_ = 2.19 p = 0.063). T-tests showed that this effect related to a lack of sensitivity to novelty within the caudate but not the IFS or IPC ROIs (Fig. 5a).

A rule-novelty contrast was then estimated for each ROI during learning from feedback (Stages 1–5 weighted as 2 1 0 − 1 − 2) and these data were examined in three separate one-way repeated measures ANOVAs with the condition ROI (6). There were significant effects of ROI within the AIFO network (F_(5,65)_ = 10.07 p < 0.001) and the IFS network (F_(5,65)_ = 4.085 p = 0.003) but not the LFPC (F_(5,65)_ = 0.908 p = 0.481). T-tests showed that, unlike learning from instruction, there were significant effects of novelty within all IFS network ROIs including the caudate bilaterally. There were also significant effects of novelty within the AIFO network ROIs with the exception of the TPJ (Fig. 5b).

### Network activations during feedback

Parameter estimates for the negative feedback events were extracted from each ROI. These data were analysed in a GLM with conditions Network (3) ∗ ROI (6). There were significant effects of Network (F_(2,26)_ = 3.601 p = 0.042) and Network ∗ ROI (F_(10,130)_ = 8.199 p < 0.001). When T-tests were conducted on data averaged across all nodes of each network there were significant effects of Feedback within all three networks (AIFO t = 4.74 p < 0.001; IFS t = 4.00 p = 0.002; LFPC t = 2.67 p = 0.019). ANOVAs focused on each network showed significant main effects of ROI within the AIFO network (F_(5,65)_ = 12.641 p < 0.001) and the LFPC network (F_(5,65)_ = 4.512 p < 0.001) but not the IFS network (F_(5,65)_ = 2.103 p = 0.076). Post-hoc t-tests examining the basis of the differences showed significant effects for all ROIs with the exception of the left TPJ, the left parietal cortex and the bilateral frontopolar cortices (Fig. 5c). Examining parameter estimates for positive feedback events using a network ∗ ROI GLM of the same design showed no significant main effects or interactions (all p > 0.1). T-tests conducted on each individual ROI also showed no significant effects of positive feedback within any ROI even at the liberal uncorrected and one-tailed threshold of p < 0.05. Therefore, effects of positive feedback were not examined further in this study.

### Analysis of phase synchrony

**Fig. 6:**
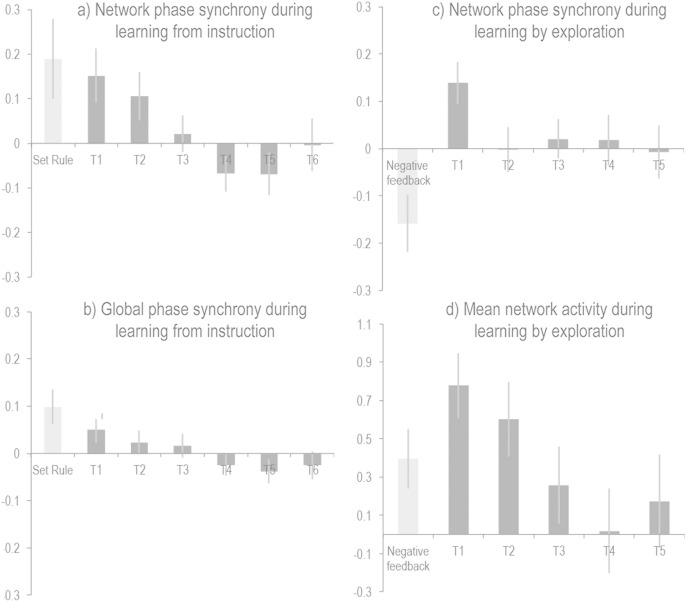
a) In Study 1, analysis of ROI phase synchrony showed an increase in network functional connectivity when processing novel rules. b) This effect was also evident when examining phase synchrony across all voxels within the brain. (Error bars report the standard error of the mean). c) In contrast to learning from instruction, there was only an increase in network phase synchrony at the first stage during learning by exploration with feedback. Unexpectedly, there was also a decrease in network phase synchrony during the reception of negative feedback. d) In Study 2, the phase synchrony effects were uncoupled from the activation magnitude effects during learning by exploration with feedback. (Error bars report the standard error of the mean).

Phase synchrony analyses were conducted between all ROIs to determine whether the effects of learning on activation magnitudes were accompanied by changes in the global functional connectivity of LFC networks (Hellyer et al., 2014, Parkin et al., 2015). In the analysis of learning from instruction, the psychological predictor functions, movement parameters and constant term were regressed together onto the global phase synchrony timecourse of the network ROIs. The resultant parameter estimates were examined at the group level using repeated measures ANOVA with Stages 1–6 as the condition. There was a significant main effect (F_(5,80)_ = 6.12 p < 0.001), which was driven by greater phase synchrony in the earlier learning stages followed by a smooth downwards curve as rules became familiar (Fig. 6a). Heightened phase synchrony was also evident when rules were being defined (t = 2.11 p = 0.05). Repeating the analyses with the phase synchrony timecourse calculated across data from all voxels within the brain showed a significant main effect of Stage (F_(5,80)_ = 3.24 p = 0.01) and a significant effect of rule definition (t = 2.67 p < 0.05) (Fig. 6b). Therefore, there was a global increase in brain functional connectivity when novel rules were being learnt from instruction.

ROI data during learning from feedback were analysed in the same manner. One outlier value was winsorised as it was > 2.5 SDs from the mean. There was a significant effect of Novelty (F_(4,52)_ = 2.622 p = 0.045); however, heightened network synchrony was only evident in the first learning stage (t = 3.134 p = 0.008) (Fig. 6c). Furthermore, negative feedback events were associated with a significant decrease in network synchrony (t = − 2.618 p = 0.021). Notably, this network desynchronisation occurred in conjunction with a significant global increase in network activation (Fig. 6d), thereby uncoupling the phase and magnitude effects.

### Psychophysiological interactions

**Fig. 7:**
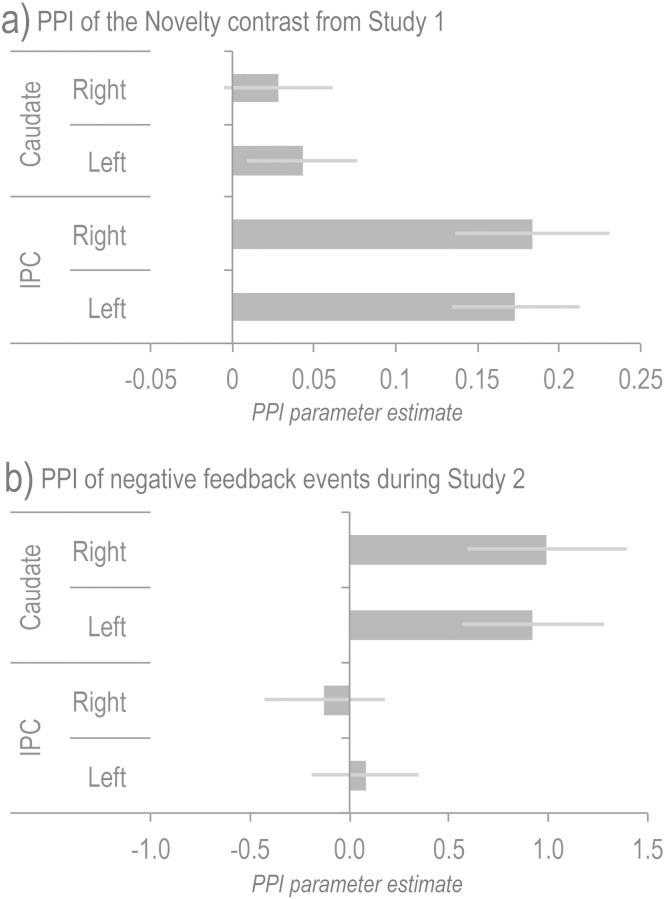
a) Psychophysiological interaction of the IFS ROI timecourse with the rule novelty contrast showed greater functional connectivity with the IPC but not the caudate ROIs during learning by instruction. 7b) Psychophysiological interaction of the IFS ROI negative feedback event timecourses showed a significant increase in function connectivity with the caudate but not the IPC ROIs during learning by exploration with feedback. (Error bars report the standard error of the mean).

As described above, the contrast of rule novelty during learning from instruction showed no significant increase in caudate activation levels during early relative to late learning stages (Fig. 5a); however, it was still possible that the region could have had stronger functional connectivity with the IFS during those early stages. This possibility was tested using PPI analysis, which provides a focused method for examining task-related changes in functional connectivity between pairs of brain regions (Friston et al., 1997). A PPI model was constructed with the Novelty contrast (Stages 1–6 weighted as 3 2 1 − 1 − 2 − 3) as the psychological predictor, the average of the IFS ROI timecourses as the physiological predictor and their interaction as the PPI. T-tests of the resultant parameter estimates showed no significant PPI effect within the caudate ROIs (both p > 0.2 Fig. 7a). For comparison, robust PPIs were evident when regressing the same set of predictor functions onto the IPC ROI timecourses (both p < 0.001 2-tailed), which form part of the same IFS network.

Applying the same PPI analysis to data during learning by exploration with feedback also showed no significant effect of Novelty on functional connectivity between the IFS and caudate. However, when a PPI model was generated using the timecourse of negative feedback events interacted with the IFS activation timecourse, there was a significant PPI effect (left t = 2.625 p = 0.021 right t = 2.496 p = 0.027 Fig. 7b). For comparison, there was no significant effect when regressing the same set of predictor functions onto the IPC ROI timecourses (left t = 0.288 p > 0.5 right t = − 0.377 p > 0.5). Thus, although there was a global decrease in functional connectivity across the LFC networks during the reception of negative feedback, the IFS-caudate connections showed a significant increase. Furthermore, there was a double dissociation between corticocortical and corticostriatal connections within the LFC network, with the former being sensitive to rule novelty and the latter to negative feedback.

### Dynamic causal modelling

Learning from instruction generated significant effects in terms of the activation magnitudes, phase synchrony and PPIs of LFC networks; therefore, a pertinent question was how learning-related changes in network connectivity propagated across the LFC nodes. To address this question, we compared eight dynamic causal models (DCMs) that instantiated alternative hypotheses regarding LFC functional organisation. The analyses applied standard bilinear and non-stochastic DCMs, which included directional functional connectivities between nodes (A matrix), psychological modulators of the strengths of the directional connections (B matrix) and driving inputs of psychological events (C matrix) to network nodes. Models were fitted to the activation timecourses extracted from the AIFO, IFS and LFPC ROIs, collapsed across hemisphere, to form three nodes. Caudate ROIs were not included in this analysis because they showed neither task nor learning related activations or connectivities when performing discriminations in Study 1.

**Fig. 8:**
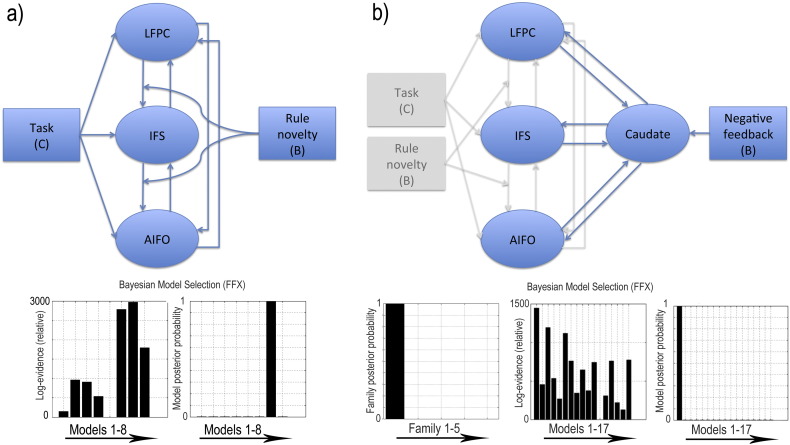
a) In Study 1, Bayesian model selection with fixed effects analysis favoured a dynamic causal model in which rule novelty modulated top-down connections from the LFPC to the IFS and from the IFS to the AIFO. b) In Study 2, Bayesian model selection with fixed effects analysis favoured the family of models in which negative feedback was a driving input. Model 1, in which the driving input was via the caudate ROI provided the best explanation of the data.

The timecourses for the Task and Novelty contrasts formed the psychological inputs to the DCMs. The hypotheses under investigation pertained to how task-related interactions between LFC regions varied as a function of learning stage. Therefore, the Task timecourse was applied as a driving input to all three nodes in all eight models and the three nodes had reciprocal connections. Novelty formed the modulatory input and differed with respect to the targeted connections. Three models represented the hypothesis that changes in functional connectivity during learning from instruction were driven by one LFC sub-region. Therefore, Novelty modulated both efferent connections from just one of the nodes. Another two models represented the hypothesis that learning has a general effect on network connectivity with no specific LFC source. Therefore, either all of the between node connections or none of those connections were modulated by Novelty. Finally, three models represented the hypothesis that the learning effects are propagated via a hierarchical arrangement of LFC sub-regions. Therefore, the AIFO → IFS and IFS → LFPC connections were modulated in a posterior-to-anterior model, LFPC → IFS and IFS → AIFO connections were modulated in an anterior-to-posterior model, or both sets of connections were modulated together. Bayesian model selection with fixed effects analysis determined which model provided the best balance between model complexity and fit to the fMRI timecourses. The results favoured the three hierarchical models and the anterior–posterior model in particular (Fig. 8a). This model accounted for 17% of the variance on average, substantially above the proposed criteria of 10% for good DCM model fit (Stephan et al., 2010).

The analyses of activation magnitudes and PPIs had demonstrated that learning by exploration with feedback involved additional striatum activation and frontostriatal interactions that were not evident when learning from instruction. Therefore, 17 dynamic causal models were compared to determine the causal basis of these effects. All models built on the anterior–posterior architecture identified in the analysis of Study 1. They included the caudate ROIs as a fourth node and the negative feedback events as an additional psychological timecourse. The hypotheses under examination pertained to the target of negative feedback within this network. Therefore, all models had bidirectional connections between the caudate and the other three nodes but they varied with respect to the target of the feedback timecourse.

The models were analysed in five families to compare hypotheses at the complementary levels of like and individual models. A family of four models represented the hypothesis that negative feedback has a driving as opposed to modulatory impact on the LFC networks. Therefore, feedback had a driving (C matrix) input to either the caudate or one of the three LFC nodes. An individual model represented the null hypothesis that negative feedback has a negligible impact on LFC networks. Therefore, feedback had no driving or modulatory targets. Another family of three models represented the hypothesis that feedback modulates interactions between the caudate and just one of the LFC regions. Therefore, feedback targeted (B matrix) bidirectional connections between the caudate and one or other of the LFC nodes. A fourth family of three models represented the hypothesis that feedback modulates multiple connection pathways between the caudate and LFC in parallel. Therefore, feedback targeted all connections to, from, or bi-directionally with the caudate. The final family of six models represented the hypothesis that negative feedback modulates a specific directional connection between the caudate and the LFC. Therefore, feedback targeted one of the individual afferent or efferent connections between the LFC nodes and the caudate. Bayesian model selection with fixed effects analysis clearly favoured the first family of models and the model in which negative feedback was a driver of caudate activation in particular (Fig. 8b). This model accounted for 21% of the variance.

## Discussion

This study provides novel insights into the mechanisms and network interactions that support intentional learning. We found that the LFC networks activated *en masse* and in synchrony when simple discrimination rules were initially being learnt. They then transitioned towards a low activation and asynchronous state when those same discriminations were routine. This was the case even when the rules were unambiguous, having been defined based on explicit instruction at the start of the learning block. These results accord with the hypothesis that LFC networks work together to support a temporary internal program that enables tasks to be performed at the earliest stages of learning.

### LFC networks form a functional hierarchy

The LFC networks were not recruited in a uniform manner dependent on the overall level of cognitive demand; instead, they were engaged in different combinations and interacted in alternative connectivity states across the stages and types of learning. For example, when analysing the magnitude of activations, it was evident that the LFC networks disengaged from the task in a step-wise manner as learning progressed, with the LFPC showing the most rapid decrease in activation and AIFO showing the slowest decrease. These results accord with the hypothesis that LFC networks conform to a functional hierarchy. The analyses of dynamic causal models tested this hypothesis in a more formal manner by comparing models in which different combinations of directed connections were modulated by learning stage. Bayesian selection strongly favoured the model in which the connections were modulated along a rostral to caudal axis. This rostro-caudal model replicates the network dynamics that are observed during relational reasoning (Parkin et al., 2015), suggesting that the hierarchical change in causal information flow may be common across reasoning and rule-learning tasks.

Several researchers have proposed that the human LFCs house a hierarchical system, the levels of which support increasingly high order cognitive processes. This proposal is primarily based on studies that have compared the regional activations of the LFCs during the performance of increasingly complex cognitive tasks or that characterised cognitive impairments in patients with focal LFC lesions (Badre, 2008, Badre et al., 2009, Hampshire et al., 2007, Koechlin et al., 2003, Ramnani and Owen, 2004). Notably, it has been suggested that the LFPCs sit at the apex of this hierarchy and support relational integration, whereby cognitive processes of other LFC regions are combined to form higher-order products. In accordance with this view, the LFPCs are more active during tasks that require cognitive processes to be sequenced or integrated (Vendetti and Bunge, 2014) including spatial planning (Hampshire et al., 2013, Williams-Gray et al., 2007) relational reasoning (Bunge et al., 2009, Christoff and Gabrieli, 2000, Christoff et al., 2001, Christoff et al., 2009, Parkin et al., 2015, Wendelken et al., 2008) and contingency reversal learning (Hampshire and Owen, 2006). Our results demonstrate that this hierarchy extends to include the broader functional networks that each LFC sub-region is associated with.

However, it should be noted that the discrimination rules applied in the current study involved no higher-order relations. Furthermore, the stages in Study 1 only differed according to the novelty of those rules; that is, there were no sequencing, feedback-contingency or integration demands to process. Therefore, although the LFPC network is recruited during relational integration and may make a unique contribution to such processes (Parkin et al., 2015), this is unlikely to be its only cognitive role because it is also involved in the simplest of discrimination tasks when the stimulus–response rules are novel. Instead, LFC networks may be recruited step-wise as a variety of cognitive demands increase, whilst the flow of information between them varies dependent on the type of process that they are supporting.

### Functional dissociations and interactions between frontal and striatal network nodes

The most striking dissociation was between cortical and subcortical components of the LFC networks. Specifically, frontoparietal regions were sensitive to the simplest form of intentional learning in which rules were explicitly instructed. In contrast, the ventral caudate regions only showed significant activation during the task in which rules were derived via a process of trial-and-error; they were particularly active during the reception of negative feedback and showed no significant activity during the reception of positive feedback. This pattern of results accords with complementary roles for cortical and sub-cortical components of the LFC networks in maintaining/applying and updating the internal task program respectively.

The analysis of connectivity in Study 2 provided further insights into the interactions between frontal and striatal components of the LFC networks. For example, during negative feedback there was heightened activation and decreased functional connectivity across the cortical nodes of the LFC networks. A tentative explanation for this finding is that the exploration of alternative discrimination rules is a chaotic process that requires the destabilisation of one internal program to allow the formation of another. This process may be reflected at the network level by a desynchronisation and resynchronisation of the frontoparietal regions that represent the internal program. The PPI analyses support the view that the caudate-IFS connections played a role in this process. Specifically, the global decrease in network synchrony during negative feedback was accompanied by a significant increase in functional connectivity between these regions. Thus, cortical and striatal connections of the IFS network were doubly dissociated by their sensitivities to learning stage and learning type.

The striatum comprises a functionally heterogeneous set of structures. The regions of interest analysed here were centred on mid-ventral sub-regions of the caudate nuclei and were localised by their functional connectivity profiles in another study (Parkin et al., 2015). These ROIs were proximal to the peak activation coordinates within the striatum during negative feedback. As demonstrated by the analyses of dynamic causal models, these regions up-regulate the LFC networks during the reception of negative feedback, thereby promoting exploratory behaviours. Notably though, increases in the activation of the ventral striatum have been reported to relate to expectancy violation during feedback (Glascher et al., 2010, O'Doherty et al., 2003), that is, as opposed to the feedback valence per se. In accordance with these findings, we have also previously reported activation within a similar region during the control condition of a novel reversal-learning task, when exploratory behaviours were cued by the presentation of new stimulus sets with no prior negative feedback event (Hampshire et al., 2012a). Interestingly, caudate activation in that study was only evident in response to negative feedback events if they triggered a subsequent change in behaviour (Hampshire et al., 2012a). More broadly, increased activation and connectivity of the ventral striatum has been reported during problem-solving conditions that are not cued by negative feedback; for example, spatial planning (van den Heuvel et al., 2005) and relational reasoning (Parkin et al., 2015).

Together, these results indicate that the striatocortical circuit is involved when a temporary internal program that is used to perform the task, is modified. That is, in contrast to processing feedback or reward expectancy violations per se. As a further test of this hypothesis, we conducted an analysis of the activation within the caudate nucleus during presentation of the rule definition slide in Study 1. It is important to note, that there were few of these events in the study; therefore, we have limited power to examine this condition. Nonetheless, it is informative that this was the only condition in Study 1 to produce significant caudate activation (left t = 1.88 p = 0.03; right t = 2.40 p = 0.009). This result provides tentative evidence that this region of the ventral striatum is involved when the internal task program is being formed/updated, even under conditions where there is no feedback.

### Non-monotonic learning effects

Unexpectedly, the learning-related decreases in response times in both studies were not monotonic. During learning from instruction, the early minimum occurred in the third of the thirty-second stages. The non-monotonic effect was not evident in the magnitude or phase synchrony timecourses; however, it coincided with the stage at which network phase synchrony ceased to be greater in the contrast of discriminations vs. fixation. We therefore suggest that these behavioural effects are an emergent property of the transition from intentionally controlled to routine modes of task performance. Put another way, once the task becomes routine, it is no longer necessary to actively maintain a model for performing it and the LFC networks disengage. The subsequent gradual decline in RTs may reflect the continued consolidation of the discrimination mappings in habitual stimulus–response pathways.

During learning by exploration, the early minimum occurred sooner than that observed during learning from instruction and was less robust. One might have predicted the opposite pattern of results with the learning effects of Study 1 being delayed by the duration of the exploration phase in Study 2. However, we suggest that learning by exploration with feedback is a fundamentally different process; it engages additional striatocortical circuits and involves powerful reinforcement learning mechanisms. The requirement for these additional processes may initially slow RTs but they may also work to accelerate the consolidation of new behaviours. In accordance with this view, the analysis of activation magnitudes not only showed additional recruitment of regions within the striatum during the early stages of learning, there were also significant learning effects within the AIFO network. Moreover, the RT minimum in Study 2 again coincided with the stage at which LFC network phase synchrony was no longer greater for discriminations relative to fixation. A future challenge will be to determine how these non-monotonic RT effects emerge from the shifting balance between the neural systems that support controlled and routine modes of behaviour.

### Summary

Our results support the hypothesis that the neural components supporting behavioural-control comprise a hierarchical set of networks that include nodes within the lateral frontal cortices. These networks are active and synchronous when novel tasks are being intentionally processed; however, they are dissociable by their sensitivities to the stage and type of learning. Their directed connectivity varies asymmetrically and hierarchically across different stages of the learning process. Furthermore, when negative feedback indicates the need to initiate exploratory behaviour, there is an increase in driving inputs from the caudate. A future research question is whether directional network interactions always conform to this hierarchy, or whether the lateral frontal cortex is engaged in a diverse repertoire of configurations to support different cognitive demands. Another question for a larger scale cohort study is whether these network dynamics underpin individual differences in learning ability or adaptability. From a clinical-translational perspective, we have reported that the functional connectivity of LFC networks is impacted in patients who suffer sports-related traumatic brain injuries (Hampshire et al., 2013) and have observed that patients with Parkinson’s disease, who suffer abnormal striatum function, show slowed acquisition of contingency reversal learning paradigms (Williams-Gray et al., 2008). Therefore, a sensible future direction is to determine whether the connectivity effects observed here during simple intentional learning can provide clinical-diagnostic markers in populations that suffer from cognitive impairments.
